# Phenotypic Diversification Is Associated with Host-Induced Transposon Derepression in the Sudden Oak Death Pathogen *Phytophthora ramorum*

**DOI:** 10.1371/journal.pone.0034728

**Published:** 2012-04-18

**Authors:** Takao Kasuga, Melina Kozanitas, Mai Bui, Daniel Hüberli, David M. Rizzo, Matteo Garbelotto

**Affiliations:** 1 Crops Pathology and Genetics Research Unit, United States Department of Agriculture–Agricultural Research Service, Davis, California, United States of America; 2 Department of Environmental Science, Policy, and Management, University of California, Berkeley, California, United States of America; 3 Department of Plant Pathology, University of California Davis, Davis, California, United States of America; University of Sydney, Australia

## Abstract

The oomycete pathogen *Phytophthora ramorum* is responsible for sudden oak death (SOD) in California coastal forests. *P. ramorum* is a generalist pathogen with over 100 known host species. Three or four closely related genotypes of *P. ramorum* (from a single lineage) were originally introduced in California forests and the pathogen reproduces clonally. Because of this the genetic diversity of *P. ramorum* is extremely low in Californian forests. However, *P. ramorum* shows diverse phenotypic variation in colony morphology, colony senescence, and virulence. In this study, we show that phenotypic variation among isolates is associated with the host species from which the microbe was originally cultured. Microarray global mRNA profiling detected derepression of transposable elements (TEs) and down-regulation of crinkler effector homologs (CRNs) in the majority of isolates originating from coast live oak (*Quercus agrifolia*), but this expression pattern was not observed in isolates from California bay laurel (*Umbellularia californica*). In some instances, oak and bay laurel isolates originating from the same geographic location had identical genotypes based on multilocus simples sequence repeat (SSR) marker analysis but had different phenotypes. Expression levels of the two marker genes analyzed by quantitative reverse transcription PCR were correlated with originating host species, but not with multilocus genotypes. Because oak is a nontransmissive dead-end host for *P. ramorum*, our observations are congruent with an epi-transposon hypothesis; that is, physiological stress is triggered on *P. ramorum* while colonizing oak stems and disrupts epigenetic silencing of TEs. This then results in TE reactivation and possibly genome diversification without significant epidemiological consequences. We propose the *P. ramorum*-oak host system in California forests as an ad hoc model for epi-transposon mediated diversification.

## Introduction

The growing number of sequenced genomes reveals that a large fraction of plant pathogenic fungal (including oomycetes) genomes are composed of transposable elements (TEs) [1], [2], [3], [4]. In some fungal genomes, TEs are often not distributed evenly along chromosomes, but rather form clusters with fast evolving genes that are likely involved in host-pathogen interactions. Examples include host-specificity genes in *Magnaporthe grisea* [5], *Fusarium* spp. [2] and *Verticillium* spp. [1], [2], and effector genes in *Phytophthora infestans* [3], [4]. In these examples, TEs are postulated to be involved in host-specialization and speciation of pathogens. In contrast in the powdery mildew pathogen, *Blumeria graminis*, TEs are distributed throughout the genome with no evidence of clustering [6]. The expansion of TEs and genome size detected in *B. graminis* is thought to coincide with evolution to obligate biotrophy. In both examples, TEs are associated with the emergence of evolutionarily novel lineages characterized either by a host switch or by a change in trophic behavior.

The emergence of such lineages could be considered examples of “punctuated equilibrium” where evolution proceeds through bursts of rapid morphological change and speciation followed by long term stasis [7]. Zeh and coworkers [9] have proposed the “epi-transposon hypothesis” in which TEs play a major role in punctuated equilibria. TEs are highly mutagenic, but their expression and mobility are epigenetically silenced in eukaryotic cells [8]. According to the epi-transposon hypothesis, physiological stress, associated with major climatic change or invasion of new habitats, may disrupt epigenetic silencing, resulting in TE reactivation [9]. Mobilized TEs then rapidly restructure the genome, alter gene expression patterns and mutate gene structures. This enables populations to colonize new adaptive peaks and leads to rapid morphological change and speciation [9]. There have been several examples described of concordant timing between bursts of transposition or TE extinction and speciation (reviewed in [10]). These findings, mostly in vertebrates, were made through comparative genomics of species diverged millions of years ago. Because of this, the actual evolutionary process occurring on geological time scales has not been witnessed.

Due to short generation times, host jumps, and adaptation, diversifications of plant pathogens and microbes are events more easily observable in our lifetime. We can hypothesize that drastic phenotypic and/or genomic alterations due to epi-transposon hypothesis may be observable in pathogens that are engaging in novel plant-pathogen interactions. Parker and Gilbert [11] summarized three scenarios for the emergence of novel plant-pathogen interactions. The first and most documented scenario is that of pathogens being introduced to new habitats where susceptible plants are present leading to emergent diseases. Classic examples include chestnut blight [11], Dutch elm disease [12], and white pine blister rust [13]. In the second scenario, a novel host-pathogen interaction is established between a newly introduced plant species and a local pathogen. Examples of this interaction include the soybean-*Phytophthora sojae* pathosystem in North America [14], [15], [16] and the Leyland cypress-*Seiridium cardinale* pathosystem in California [17]. The third scenario involves the interaction between originally co-evolved hosts and pathogens that are separated and then re-united in a novel environment. The most famous example of this last scenario is exemplified by potato and the potato late blight pathogen, *Phytophthora infestans* [18], [19].

In the first scenario, a newly introduced plant pathogen will face both a novel environment and novel host species while generally undergoing a very significant population genetic bottleneck. However, despite an initial genetic bottleneck, there is evidence of rapid phenotypic diversification in some introduced plant pathogens. For example, *Phytophthora cinnamomi*, a well-studied exotic root-rot pathogen of numerous woody host plants, displays a large phenotypic diversity within clonal lineages in areas where it has been introduced [20], [21]. Although, genetic variation generated by mitotic recombination explains some of this phenotypic variation [22], we hypothesize that involvement of TE-mediated diversification may be a likely candidate that may explain variability in the absence of mutation or mitotic recombination.

*Phytophthora ramorum*, the causal agent of the forest disease “sudden oak death” (SOD), is a recent example of an exotic pathogen [23] also displaying extensive phenotypic variation within a clonal lineage. This pathogen was unknown before it was observed to cause diseases of a number of host species in the mid-1990s in Europe and California. *P. ramorum* is a generalist pathogen which causes two types of plant disease: trunk girdling lesions leading to death of the entire host (known as SOD) and nonlethal leaf and twig infections with a final outcome ranging from a foliar disease to a progressive dieback of infected hosts (known as ramorum blight) [23]. SOD has been primarily described from oaks (*Quercus* spp.) and other members of Fagaceae, while ramorum blight is widespread among members of over 45 plant genera. While *P. ramorum* in North America displays a large variance in virulence, isolates of the pathogen from a single host show similar virulence levels on unrelated hosts thereby supporting a lack of host specialized sub-populations [24].

In California, *P. ramorum* produces copious sporangia (infectious spores) on the leaves of California bay laurel (bay laurel, *Umbellularia californica*), and to a lesser degree on leaves and twigs of tanoak (*Notholithocarpus densiflorus*) [25], [26], [27]. In contrast, studies have shown that sporangial formation does not occur on infected trunks of coast live oak (oak, *Quercus agrifolia*) and tanoak [25], [26], [27]. Therefore, transmission of the pathogen between oak is insignificant [28]. In forests, oak can be considered an epidemiological dead-end host for *P. ramorum* while, bay laurel is likely the most important reservoir for inoculum of the pathogen [27], [28].

Within its introduced range, *P. ramorum* only propagates clonally [29]. To date, three distinctive clonal lineages have been identified in Europe, California, Oregon, Washington State, and British Columbia [28], [29], [30]. In California and Oregon forests, only the clonal lineage NA1 is present [28]. Genetic diversity within NA1, measured by AFLP and multilocus SSR markers, is limited [28], [29], [31], [32], yet the pathogen exhibits a large variation in colony morphology and virulence [24], [33], [34]. In particular, we have noticed that *P. ramorum* isolates originating from oak frequently do not grow well following subculturing and long-term laboratory storage. Archival isolates of oak in our culture collection are more likely to die over time than those originating from other host species (Table S1). This observation is in contrast to isolates originating from foliage of various species (e.g., bay laurel or *Rhododendron* spp.). We hypothesized that NA1 is undergoing rapid diversification and phenotypic variation within the lineage mainly due to regulation of genes, rather than structural difference in the genes [35]. Investigating phenotypic diversification in *P. ramorum* is thus an opportunity to understand some of the mechanisms associated with the accelerated evolution of an exotic pathogen in a new environment. The objective of this work was to evaluate phenotypic and transcriptional diversities within the *P. ramorum* NA1 clonal lineage.

## Results

### Variation in virulence, early senescence, and colony morphology

As described by Brasier and coworkers [33], Californian NA1 isolates of *P. ramorum* show a large variation in growth rate and colony morphology (Fig. S1) including a majority of ‘wild type’ (*wt*) isolates and a minority of ‘non-wild type’ (*nwt*) isolates characterized by irregular growth patterns. Some isolates occasionally showed early senescence (i.e. failure to grow in culture).

In order to evaluate the possibility of association between colony phenotypes, geography and originating host species, we selected 45 isolates obtained in equal numbers from three California counties (Santa Cruz, Monterey and Sonoma) and three hosts (bay laurel, tanoak, and coast live oak), and two additional reference isolates (set 1 in Table 1, Table S2 for details), for a total of 47 isolates. When these isolates were analyzed using 7 polymorphic SSR markers, geographical differentiation was detected among the three California counties (Table S3, AMOVA, p<0.001), but no differentiation was detected among isolates originating from the three host species (Table S4). These results were consistent with previous studies describing the presence of genetic structure among populations from different sites without any clear host-association [24], [28], [32]. Among these 47 isolates, 15 exhibited early senescence in at least one of four replicates, while 13 isolates displayed *nwt* colony morphology. Both traits were significantly more common in isolates from oak than in isolates from other hosts (i.e. bay laurel and tanoak, Fisher exact test p = 0.0003 and 0.01 for morphology and early senescence, respectively), but county of origin had no effect on either trait (Table 2).

**Table 1:** Summary of Californian isolates of *Phytophthora ramorum* used in this study^1^.

No. of isolates	Source^1^	County	Collection year	Note^2^
**set 1**				
5	tanoak	Monterey	2004	
5	tanoak	Santa Cruz	2004	
5	tanoak	Sonoma	2002	Pr-35
5	coast live oak	Monterey	2000–2004	BS-92
5	coast live oak	Santa Cruz	2000–2004	Pr-16
5	coast live oak	Sonoma	2000–2001	
5	California bay laurel	Monterey	2004	
5	California bay laurel	Santa Cruz	2004	HC67-22, HC73-5
5	California bay laurel	Sonoma	2001–2002	Pr-177, Pr-240
1	coast live oak	Marin	2001	Pr-102
1	*Rhododendron catawbiense*	Santa Cruz	2000	Pr-52
**set 2**				
1	tanoak	Marin	2000	
1	tanoak	Monterey	2000	
1	tanoak	Santa Cruz	2000	
6	coast live oak	Marin	2000–2007	
1	coast live oak	Napa	2000	
8	coast live oak	San Mateo	2001–2009	MK106, MK558, MK516a, MK516d
7	coast live oak	Sonoma	2000–2009	
1	canyon live oak	Humboldt	2005	
8	California bay laurel	Marin	2001–2007	
1	California bay laurel	Napa	2001	
10	California bay laurel	San Mateo	2008–2009	MK79j, MK548, MK649a, MK649b
1	California bay laurel	Santa Cruz	2001	
5	California bay laurel	Sonoma	2001–2007	
2	California huckleberry	Marin	2001	
1	stream water	Humboldt	2007	Pr-514 EU1 genotype

1 Table S2 for detailed information for individual isolates. Scientific names are: tanoak, *Notholithocarpus densiflorus*; coast live oak, *Quercus agrifolia*; canyon live oak, *Quercus chrysolepis*; California bay laurel, *Umbellularia californica*; California huckleberry, *Vaccinium ovatum*.

2 Isolates described in the main text are shown.

**Table 2:** Association of colony phenotypes and *P. ramorum* groups.

Isolate grouping	obs or exp^1^	Phenotypes^2^		p-value^3^
**Colony phenotypes**		***nwt***	***wt***	
non-senescence	obs	5	27	
	exp	8.9	23.1	
senescence	obs	8	7	1.31×10^−2^
	exp	4.1	10.9	
				
**Geographical origin**		***nwt***	***wt***	
Monterey	obs	5	10	7.28×10^−1^
	exp	4.1	10.9	
Santa Cruz	obs	2	14	1.68×10^−1^
	exp	4.4	11.6	
Sonoma	obs	5	10	7.28×10^−1^
	exp	4.1	10.9	
**Originating hosts**		***nwt***	***wt***	
tanoak	obs	2	13	1.75×10^−1^
	exp	4.1	10.9	
oak	obs	10	6	2.71×10^−4^
	exp	4.4	11.6	
bay laurel	obs	1	14	3.72×10^−2^
	exp	4.1	10.9	
				
**Geographical origin**		**senes**	**non-senes**	
Monterey	obs	7	8	1.70×10^−1^
	exp	4.6	10.4	
Santa Cruz	obs	2	14	9.15×10^−2^
	exp	4.9	11.1	
Sonoma	obs	5	10	1.0
	exp	4.6	10.4	
**Originating hosts**		**senes**	**non-senes**	
tanoak	obs	3	12	3.17×10^−1^
	exp	4.9	10.1	
oak	obs	10	6	2.78×10^−3^
	exp	5.2	10.8	
bay laurel	obs	2	13	9.20×10^−2^
	exp	4.9	10.1	

1 Observed number of isolates and expected number of isolates if probabilities of each outcome are independent of colony morphology.

2 Phenotypes are nwt: non-wild type, wt: wild type, senes: early senescence, and non-senes: early senescence not observed.

3 p-values due to Fisher's exact test.

**Figure 1:**
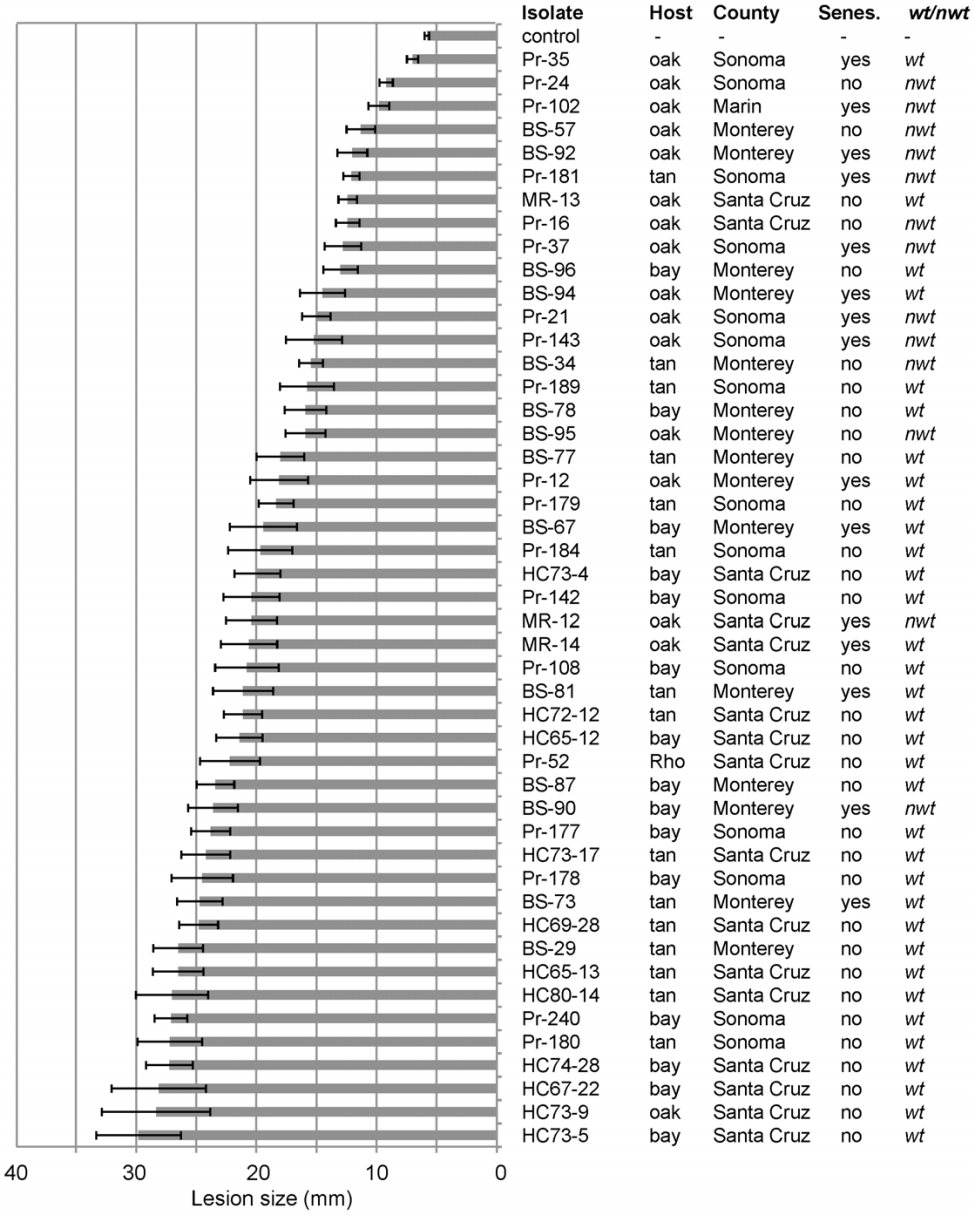
Variation in virulence of *Phytophthora ramorum* isolates on oak stems. Lesion lengths in stems (n = 10) of coast live oak were measured 16 days after inoculation. An average lesion length +/−1 standard deviation for each isolate are shown. Originating host species are: oak, coast live oak; bay, California bay laurel; tan, tanoak; and Rho, *Rhododendron catawbiense*. Originating counties of isolates are also shown. Colony morphologies are: *wt*, wild type; and *nwt*, non-wild type.

In order to study variation in virulence, these 47 isolates were then inoculated on oak seedlings. Mock inoculations of oak seedlings resulted in lesions that were significantly smaller (z-test, p = 0.00) than lesions caused by *P. ramorum* inoculations. Sizes of lesions in stems inoculated with *P. ramorum* ranged between 7 and 30 mm (Fig. 1); a Mann-Whitney U test identified significant differences among counties (p = 1.9×10^−2^) (Fig. 1, Table 3). Isolates from Santa Cruz caused lesions that on average were larger than average lesions caused by isolates from the other two counties (p = 3.6×10^−3^). Likewise, differences in average lesions caused by isolates from the three host species were significant (p = 1.1×10^−3^): Mann-Whitney U tests indicated that the lesions caused by isolates from oak were significantly smaller (p = 7.1×10^−5^), while lesions caused by isolates from bay laurel were significantly larger (p = 1.7×10^−2^) than those caused by isolates from other hosts. Additionally, *nwt* isolates and isolates with early senescent phenotype were both found to cause smaller lesions on coast live oak seedlings than *wt* and non-senescence isolates (Table 3, p = 0.0001 and p = 0.01, respectively).

**Table 3:** Association of lesion size on oak and *P. ramorum* groups according to Kruskal-Wallis rank sum test^1^.

group 1	group 2	group 3	group 4	p-value	note
**Geographical origin**					
Sonoma (15)	Monterey (15)	Santa Clara (16)	Marin (1)	1.85×10^−2^	Association between virulence and geographical locations.
Santa Cruz (16)	remainders (31)			3.64×10^−3^	Santa Cruz isolates are more virulent.
**Originating hosts** 2					
Oak (16)	Tan (15)	Bay (15)	*Rhododendron* (1)	1.09×10^−3^	Association between virulence and originating hosts.
Oak (16)	remainders (31)			7.06×10^−5^	Oak isolates are less virulent.
Tan (15)	remainders (32)			1.38×10^−1^	
Bay (15)	remainders (32)			1.66×10^−2^	Bay laurel isolates are more virulent.
**Colony phenotypes**					
senescence (15)	non-senescence (32)			1.10×10^−2^	Senescence isolates are less virulent.
*wt* (34)	*nwt* (13)			1.11×10^−4^	*nwt* isolates are less virulent.

1 47 isolates in set 1 in Table 1, which include two additional isolates Pr-102 from oak in Marin County, which was used for genome sequencing and Pr-52, a widely used isolate originally from *Rhododendron catawbiense* in Santa Clara county were used for the analysis. Numbers in parentheses indicate number of samples.

2 Oak, Tan and Bay are *P. ramorum* isolates originating from coast live oak, tanoak and California bay laurel, respectively.

### Microarray mRNA profiling

**Figure 2:**
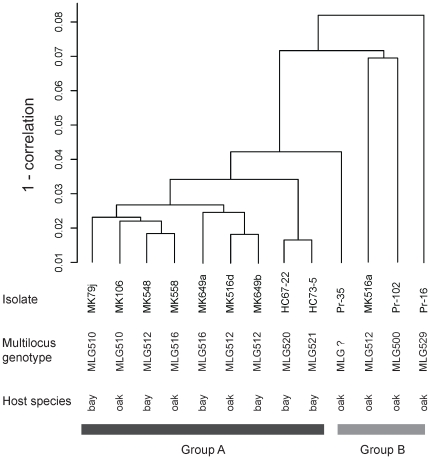
Hierarchical clustering of cDNA samples of *Phytophthora ramorum* isolates. Thirteen samples were clustered based on their expression patterns of 12,516 genes. Name of isolates, their multilocus genotypes and originating host species are shown. Group A consists of isolates that originate from either coast live oak (oak) or California bay laurel (bay), whereas all isolates in group B originated from coast live oak. Mk516a, Pr-102 and Pr-16 displayed the *nwt* phenotype. Pr-35, MK516a and Pr-102 had previously displayed the senescence phenotype but grew at the time the analysis was performed.

Thirteen isolates were used for microarray mRNA profiling (Fig. 2) including four oak isolates that either displayed nwt phenotype (Pr-16, Pr-102 and MK516a), or were growing at the time of the experiment but had earlier displayed the early senescence phenotype (Pr-102, MK516a, Pr-35), or displayed both traits (Pr-102, MK516a). The remaining three oak (MK106, MK558 and MK516d) and all of the six bay laurel isolates (MK79j, MK548, MK649a, MK649b, HC67-22 and HC73-5) had typical *wt* colony morphology and had never shown the senescence phenotype.

When global mRNA profiles were hierarchically clustered according to their expression patterns, two distinct groups could be identified. Group A displayed limited variation and included all six bay laurel isolates and the three oak isolates that have never displayed the *nwt* phenotype. Group B displayed more variability and contained the remaining isolates, all from oak (Pr-35, MK516a, Pr-102 and Pr-16), and all having displayed at some point the *nwt* and/or the senescence phenotypes. When four isolates all identified identical to MG512 by 7 variable SSRs were compared for mRNA profile, MK548 (bay laurel), MK649b (bay laurel) and MK516d (oak), all of which displayed the *wt* colony type, were found in group A, whereas MK516a (oak) displaying the *nwt* colony type, was a member of group B. In summary, colony phenotypes and originating host species were associated with global mRNA profile groups, although at different statistical significance (colony Fisher exact test, p = 0.001; host Fisher exact test, p = 0.07). Association between multi-locus genotypes and the expression profile groups was not observed (p = 1.0).

**Figure 3:**
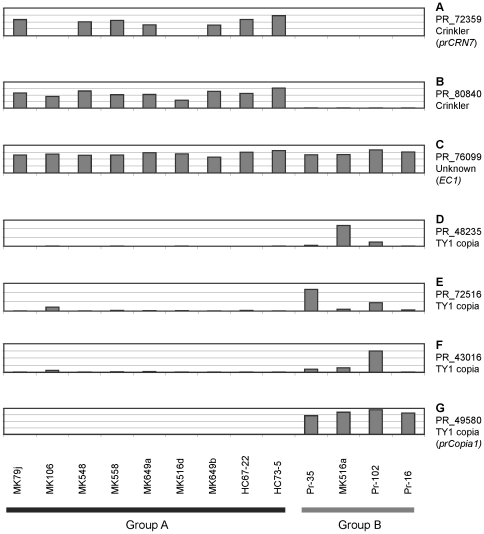
Examples of mRNA microarray profiles of *Phytophthora ramorum* genes deferentially expressed between group A and group B. Bars are estimates of relative expression levels of mRNA transcripts obtained by RMA. A gene model number and its annotation are shown for each profile. Names for corresponding qPCR markers are shown in parentheses. A) and B) CRN effector homologs. C) a hypothetical gene model PR_76099 used as endogenous control in qRT-PCR. D–G) polyprotein genes of TY1 copia retrotransposons.

In order to understand mechanisms and molecular pathways leading to these different phenotypes, we looked for genes that were differentially expressed in groups A and B. Because isolates in group B showed diverse global expression patterns, consistency in gene expression was not sought. Rather, genes with at least a five-fold difference in average expression levels between groups A and B were considered as being differentially expressed in the two groups. We identified 48 and 454 genes that were up-regulated in groups A and B, respectively (Dataset S1). Of the 48 genes overexpressed in group A, only 11 had functional annotations. Five were found to belong to the crinkler effector family [3], [36] (Fig. 3 A, B). Strikingly, a total of 297 of the 454 genes overexpressed in group B encode polyproteins from transposable elements (TEs) with diverse expression patterns (Fig. 3 D–G). Transcriptomes with elevated TEs were defined as transposon-derepressed phenotype (TDP).

### Expression patterns of TEs were diverse among oak isolates

Expression patterns of TEs varied extensively among oak isolates with TDP (Fig. 3). A large diversity of TEs has been identified in the *P. ramorum* genome (Dataset S1, Rays Jiang, personal communication). We evaluated the correlation between expression patterns and DNA sequences of TEs. A total of 262 out of 297 TE genes over-expressed in group B were grouped using BLASTCLUST into six “blast clusters” (TE1 to TE6 in Table 4). The remaining 35 TEs were found to be singletons. DNA homology was over 95% within each cluster, and below 60% among clusters. All of the 262 TEs included in one of the six clusters code for polyproteins of retrotransposons classified as TY1-copia. Of the 35 singleton TEs, 28, 7, and 1 code for Ty1-copia polyproteins, Gypsy retrotransposons, and DDE-1 DNA transposon, respectively.

**Table 4:** Association between blast clusters and mRNA profile clusters.

	mRNA profile cluster 2			
blast cluster^1^	HC1	HC2	HC3	Total
TE1	0	128	1	129
TE2	1	1	66	68
TE3	0	0	54	54
TE4	5	0	0	5
TE5	0	4	0	4
TE6	0	0	2	2
singlets	5	11	19	35
Total	11	144	142	297

1 297 TEs high in group B were clustered by BLASTCLUST.

2 Expression profiles of the 297 TEs were grouped by hierarchical clustering. Representative expression profiles are; Fig. 3-D for HC1, Fig. 3-E for HC2 and Fig. 3-F,G for HC3.

Total shows number of genes in each cluster.

Three expression clusters, namely HC1, HC2, and HC3, were identified by hierarchical clustering of the 297 TEs above (Table 4, Fig. S2). HC1 includes 11 genes with the highest levels of upregulation in isolate MK516a (e.g. PR_48235 in Fig. 3 D). HC2 includes 144 genes with high expression levels in Pr-35 and Pr-102, but otherwise inactive (e.g. PR_72516 in Fig. 3 E). HC3 contains genes with maximum expression in Pr-102 (e.g. Pr_43016 and Pr_49580 in Fig. 3 F, G).

Membership of the hierarchical clusters and blast clusters were highly correlated (Table 4). The vast majority of TEs from the same blast clusters were found in one of the hierarchical clusters of mRNA profiles. This indicates that genes in blast clusters were either coregulated and/or appeared to be coregulated due to cross-hybridization. Unfortunately, long-oligomer microarrays are incapable of distinguishing these two events. Either way, analyses based both on DNA sequence and on expression patterns concordantly indicate that TDP has multiple independent origins and does not involve only a single genomic or epigenetic alteration.

### Development of quantitative reverse transcription PCR (qRT-PCR) markers

**Figure 4:**
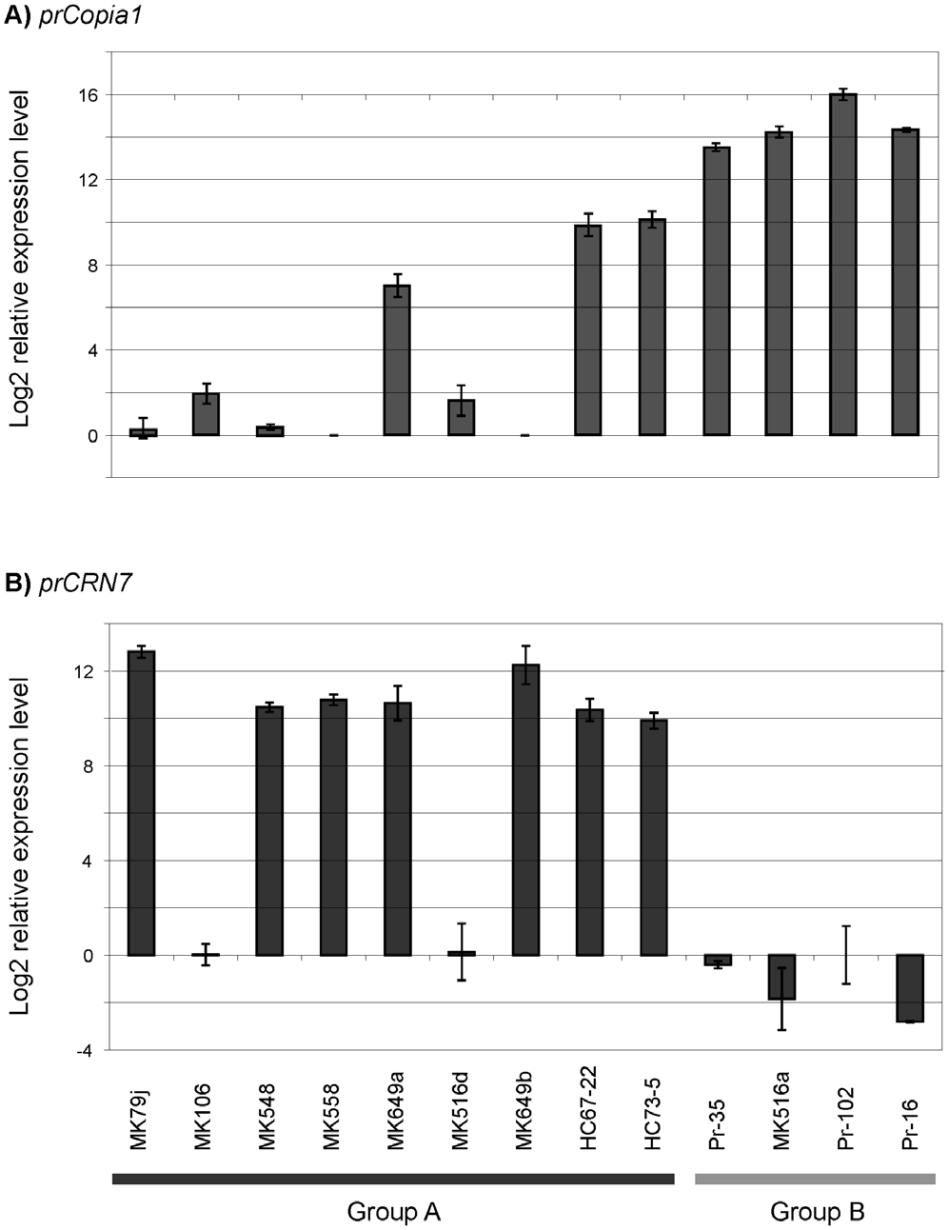
Validation of microarray data by qRT-PCR. qRT-PCR log2 fold changes (−ΔΔCT) are shown for two markers A) *prCopia1* and B) *prCRN7*. PR_76099 was used as endogenous control gene and expression levels were standardized to the genome sequence strain Pr-102. For *prCopia1*, because the standard Pr-102 had the highest expression level, its expression was offset by 16 for presentation purpose.

Two qRT-PCR markers were developed one each on a TE and a CRN gene to validate the microarray results and to differentiate isolates based on mRNA expression patterns in a high-throughput manner. These markers reproduced the trends revealed by microarray mRNA profiling. The four oak isolates in group B ranked at the top for the expression of copia-like transposon PR_49580 (hereafter termed *prCopia1*)(Fig. 4 A). While qRT-PCR detected a large variation in expression of *prCopia1* in group A, expression levels of A isolates were below the detection threshold in the microarray analysis (compare Fig. 4 A and Fig. 3 G). In agreement with microarray profiling results, qRT-PCR analysis showed high expression of the crinkler homolog PR_72359 (hereafter termed *prCRN7*) for all group A isolates but MK106 and MK516d, whereas all group B isolates showed low expression of the same gene (compare Fig. 4 B and Fig. 3 A).

### Estimation of population diversification by means of mRNA expression markers

**Figure 5:**
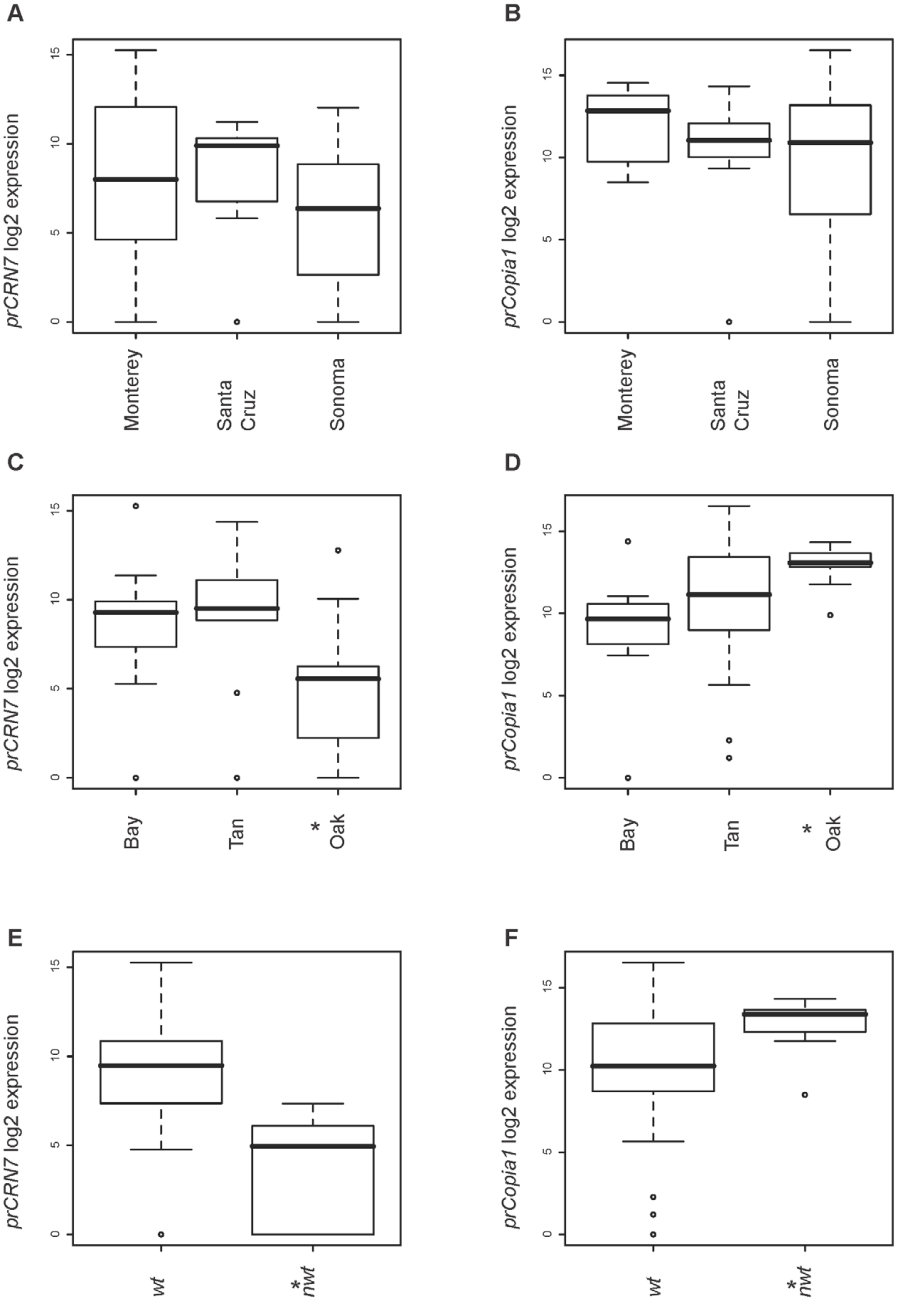
Box plot representation of *prCRN7* and *prCopia1* expression data. The matched isolates for geography and originating hosts (Table 1, set 1) are categorized into originating counties (A, B), hosts (C, D) and colony morphology groups (E, F). 25th, 50th and 75th percentile are shown with whiskers with maximum 1.5 interquartile range.

The two qRT-PCR markers *prCRN7* and *prCopia1* were finally used to evaluate potential differences due to geography or host of provenance (set 1 in Table 1). While geography had no effect on expression profiles (Fig. 5 A, B), host of provenance had a significant effect on both markers (Fig. 5 C, D). Some oak isolates, but not a single bay laurel isolate, had lower *prCRN7* expression as well as higher *prCopia1* expression. Additionally, when isolates where pooled based on colony type (i.e. *wt* vs. *nwt*) (Fig. 5, E, F), *nwt* isolates showed significantly lower *prCRN7* expression and higher *prCopia1* expression than *wt*.

When an additional 43 *P. ramorum* isolates (set 2 in Table 1) collected a few years later than the 47 isolates in set 1 were included in the analysis, similar trends were detected in mRNA profiles, independent of time since original isolation (Fig. S3). Again, varying expression patterns were seen among oak isolates, but on average they exhibited higher expression of *prCopia1* and lower expression of *prCRN7* than isolates from bay laurel and tanoak.

### Correlation between virulence and qRT-PCR results

We have shown that oak isolates were less virulent when inoculated on oak (Table 3). qRT-PCR analysis showed that in oak isolates, average *prCRN7* gene expression was low, while *prCopia1* expression was high. Expression of *prCRN7* and lesion size on coast live oak had a positive correlation (Spearman's rho = 0.498, p = 0.0004), while *prCopia1* expression and lesion size on coast live oak were negatively correlated (Spearman's rho = −0.502, p = 0.0003). No interaction effect between *prCRN7* and *prCopia1* on virulence was detected (two-way ANOVA, p = 0.1).

## Discussion

The NA1 lineage of *P. ramorum* in North American forests is known to be clonally reproducing [29], and to have been generated by the introduction of a few closely related individuals [32], yet the lineage shows diverse phenotypic variations in colony morphology [33] and virulence on host plants [24]. We have noticed that oak isolates lose viability in the laboratory more frequently than isolates from other hosts (Table S1).

In this study, we found isolates from oak were more likely to show *nwt* and early senescence phenotypes and to be less virulent on oak than isolates from bay laurel and tanoak. Elliot and coworkers [34] have also observed reduced level of virulence of *nwt* isolates on detached *Rhododendron* leaves. Multilocus SSR markers, however, failed to show genetic differentiation among isolates originating from oak, bay laurel or tanoak, and different phenotypes (*wt*/*nwt*) were observed in seven identical multi-locus genotypes (MLG 510, 512, 515, 516, 519, 525 and 527 in Table S2). For this reason, gene regulation rather than genetic polymorphism was investigated for the source of phenotypic diversity. Microarray global mRNA profiling detected derepression of TEs and down-regulation of CRNs in some of oak isolates, but never in bay isolates. Two qRT-PCR markers representing one TE and one CRN locus were subsequently developed to evaluate a larger number of *P. ramorum* isolates. Elevated expression of the TE and suppressed expression of the CRN were confirmed to be associated with *nwt* colony phenotype. Additionally this complex phenotype (morphology, virulence, and mRNA profiles) was shown to be found only among oak isolates.

### Development of TE derepressed phenotypes

Three scenarios may theoretically explain the evolution of TE-derepressed phenotypes. The first scenario postulates the involvement of infectious agents such as viruses to mobilize transposons in plants (reviewed in [37]). However, despite several attempts, no viral dsRNA has been identified in oak and bay laurel isolates belonging to the NA1 lineage [33], [38]. Only deep sequencing of the genomes or transcriptomes of oak and bay laurel isolates might allow us to safely discard the possibility of the presence of infectious agents triggering TE expression. The second scenario postulates a host-driven population diversification leading to several sub-lineages adapted to different ranges of host species. In this scenario, oak isolates with TDP would be members of a clonal sub-lineage spread from oak to oak. TDP could be a consequence of adaptation or genetic drift. Most evidence contradicts this scenario. Not only our study failed to detect genetic population diversification associated with originating hosts, but, high-density SNP genotyping using over 10,000 polymorphic sites and microarrays has been previously attempted (Tyler personal communication) and resulted in even less resolution than SSR -based analysis, upholding the lack of subgroups with clear genetic differences within the NA1 lineage. Another fact invalidating this scenario is that oak is known to be infected mostly from neighboring bay laurel [27], [39], and no oak to oak infections have ever been documented. Once this unidirectional bay laurel to oak infection process is taken into consideration, the scenario of an underlying genetic diversification of genotypes for TDP and non-TDP, albeit possible, appears quite improbable [24].

The third scenario hypothesizes that oak hosts acquire *P. ramorum* from nearby foliar hosts, and that the derepression of TEs may develop independently in each infected oak. The fact that the *nwt* phenotypes associated with TDP represent a rather varied group, with significant differences in expression profiles, yet with the same end-result of reduced fitness, strongly argues against the presence of identical undetected genetic mutations causing the change in phenotype. Rather, the diversity in expression profiles here documented among *nwt* isolates points to a general mechanism of phenotypic alteration correlated with TE derepression that may be differently modulated by timing of change and by differences in host and habitat. Molecular mechanisms underlying generation of TE derepression and its maintenance upon subsequent isolation and axenic subculturing are yet to be unraveled.

### Cause-effect relationship between alteration in global gene expression and reduction in virulence

Derepression of TEs and suppression of CRNs in axenic culture were negatively correlated to lesion size developed on oak. However, causality among these three events is ambiguous. We found TDP is associated with oak; the oak environment appears to disrupt epigenetic regulation in the pathogen and unleashes TEs. It is possible that the observed reduction in virulence may be due to epigenetic perturbation in regulatory circuits of genes required for *in planta* proliferation, and therefore the TE derepression is not the cause but simply a byproduct of epigenetic relaxation. Alternatively, the reduction in virulence may be caused by mutagenic effects of transposition; TEs can cause disruptions in gene expression by randomly inserting themselves within ORFs, often with the added consequence of causing chromosomal breaks, deletions, or chromosomal rearrangements [8]. The two phenotypes, *nwt* colony morphology and early senescence, which are both associated with TDP, could also be explained by either of the two scenarios. The phenotypic changes caused by epigenetic alteration should be reversible whereas those caused by transpositions may not depending on the extent of genomic disruption TEs have caused. Identification of genome alteration in oak isolates due to transposition and test of phenotypic reversibility by means of passage experiments of oak isolates through bay laurel leaves, are under investigation.

It is also unclear whether the observed reduction in virulence is due to reduction in CRN effectors or alternatively reduction in CRN transcripts is again a mere byproduct of changes in epigenetic status. CRN cytoplasmic effectors were originally identified from *P. infestans* transcripts encoding putative secreted peptides that elicit necrosis *in planta*, a characteristic of plant innate immunity [36]. In *P. ramorum,* 19 genes have been annotated as belonging to the CRN effector family [3]. Gene expressions of 10 CRN genes were detected by our expression microarray, and five were found to be down-regulated in oak isolates in group B. In *P. infestans*, CRN genes often cluster in the genome [3]. Obvious clustering was not observed for CRN genes in *P. ramorum* except for two CRN homologs found in close proximity. Gene functions of CRN homologs in *P. ramorum* have not been determined, but at least some of the homologs are likely cytoplasmic effectors and play an important role in pathogenicity. Shan and coworkers [40] suggested that rapid reversible changes observed in the avirulence phenotypes of some *Phytophthora* strains during asexual propagation [41], [42], could be explained by epigenetic changes in transcription levels of RXLR effector (avirulence) genes. Likewise, the change in virulence of *P. ramorum* can be explained by epigenetic changes in CRN effector homologs, which had occurred inside an oak host. Akinsanmi and coworkers [43] have observed a reduction in aggressiveness of plant pathogenic fungi after passage through alternate host species. Passage experiments such as this can also be explained by changes in expression level of effector genes due to host-induced epigenetic alteration, rather than by selection on genotypes for reduced aggressiveness. Further work is needed to decipher the role of epigenetics in host-pathogen co-evolution.

### Evolutionary implication of TE derepression

Due to accelerating rates of global trade, the rise in incidence of novel plant-pathogen interactions is inevitable. However, an epidemic outbreak caused by an exotic pathogen occurs only when the pathogen has successfully achieved both host shift and acclimatization to the new habitat. To accomplish this drastic adaptation, genetic innovations might be needed. Rapid diversification observed in exotic pathogens suggests punctuated equilibrium, suggested by some to be driven by TEs mobilized under physiological stress [9]. We observed a burst of TE expression in oak isolates of *P. ramorum* in concomitance with phenotypic alterations including colony growth abnormality and early senescence.

All published evidence so far negates the presence of host preference or adaptation of groups of genotypes within *P. ramorum*, rather we believe that the phenotypes described as associated with different hosts in this and in a previous study [24] may be tightly associated with the different epidemiological role played by different hosts. While a strong selection pressure must operate on pathogen genotypes on a transmissive host such as bay laurel, the epidemiological consequences of changes of fitness in dead-end hosts are mostly insignificant in terms of disease spread. In many respects our results parallel those from well-studied zoonotic or vector-borne diseases that include both transmissive epidemiologically relevant hosts and dead-end hosts. In zoonotic diseases, genetic and epigenetic changes providing an increased transmission of the causal agent are normally detected at high frequencies only in transmissive and not in dead-end hosts [44]. Genotypes bearing mutations or changes that lead to a loss of transmission are bound to be eliminated on a transmissive host [45], conversely, such genotypes may be found in non-transmissive dead-end hosts [46], [47]. This is particularly true of ‘spill over’ diseases like SOD, where outbreaks on a new host are not the result of novel host-adaptation, but are rather the result of the presence of a generalist disease agent affecting a transmissive and a dead-end host that happen to be in close proximity [48]. Our study may be one of the first documenting a similar process for a plant disease: TE derepression, leading to reduced isolate viability, was documented predominantly in isolates from dead-end oak hosts, while expression of CRN genes, a group of genes known to be involved in pathogenicity, was significantly up-regulated in isolates from transmissive bay laurel.

Zeh and coworkers [9] argued that in small populations, high TE-based mutation rates relaxed selection on deleterious insertions, and that the capacity of TEs to yield novel coding and regulatory elements promotes evolvability and maintains heritable variation. The same authors also pointed out that TE mobilization may frequently result not in evolutionary innovation but population decline and extinction. This situation seems to mirror the isolates of *P. ramorum* with TDP, which are phenotypically unstable and less virulent on oak. However, due to copious sporulation on foliage of bay laurel, *P. ramorum* infection continues to spread to oak and consequently, *P. ramorum* individuals showing TDP continue to be generated. We need to be vigilant to monitor evolutionary trajectory of *P. ramorum* in oak as the phenotypic variability observed may be determined by complex genetic processes that could allow for the generation of variants that may be able to sporulate on oak.

Finally, while the evolutionary consequences of TDP are uncertain for *P. ramorum*, this mechanism may be of paramount importance to explain the process leading to the documented survival of some infected oak trees [49]. Thus, TDP and any other mechanism associated with *nwt* phenotype in *P. ramorum* may have a pivotal evolutionary consequence on all native California oak species susceptible to SOD and on tanoak. Additionally, a further understanding of the mechanisms leading to the *nwt* phenotype in the pathogen may provide insights on novel approaches to control this devastating emergent forest disease.

## Materials and Methods

In this research, Californian *P. ramorum* isolates were 1) assigned to multilocus genotypes, and 2) compared for virulence on coast live oak, and colony phenotypes. Subsequently, the association between the above characters and originating host species was evaluated by means of microarray global mRNA profiling. After that, qRT-PCR markers were selected based on differential expression between isolates originating from coast live oak and California bay laurel. All permits to work with *Phytophthora ramorum* in the lab and the field in California have been secured from the California Department of Agriculture (Permit No. 2201). Collecting permits have also been obtained for all sites where field plots have been established. These include California State Parks (blanket permit for all state parks), National Park Service (Redwood National Park and associated North Coast State Parks, Pt. Reyes National Seashore), Marin Municipal Water District, Monterey Regional Parks District, East Bay Regional Parks, Big Sur Land Trust, and Mid-Peninsula Open Space.

### Isolates and culture conditions

Table 1 (Table S2 for details) lists the isolates of *P. ramorum* used in this study. A total of 101 Californian *P. ramorum* isolates was examined in this study. 96 out of the 101 isolates came from infected plant hosts, including tanoak, coast live oak or California bay laurel. Multilocus genotypes (MLGs) were determined for 91 isolates by scoring seven SSR loci known to be variable within the NA1 lineage of this pathogen. Two additional isolates were obtained not from naturally infected native plants, but from an infested stream (Pr-514) and from an infected *Rhododendron catawbiense* in a production nursery (Pr-52). Cultures were maintained on small plugs of 20% clarified V8 Juice with 1.5% agar (1× CV8A) [50] submerged in water at 20°C.

In order to study the potential effects of region and of host of origin on genetic structure, virulence and colony morphology, we employed a subset of 45 isolates from three counties (Santa Cruz, Sonoma, and Monterey). In each county, five isolates were obtained from tanoak, five from oak and five from bay laurel (Table 1, set 1). SSR alleles were analyzed by AMOVA to determine the presence of significant genetic structuring among counties and among hosts on this set of isolates.

Finally, in order to identify differential gene expression between bay laurel and oak isolates while reducing mRNA expression noise due to potential genome divergence, four pairs of identical MLGs (two pairs had identical MLGs, a total of three unique MLGs), isolated in the same site but from different hosts (bay laurel vs. oak) were analyzed through microarray mRNA profiling (Fig. 2).

### DNA extraction and SSR analysis

Isolates were grown on 7 ml 1× CV8A in a 6 cm diameter Petri dish (Bioscience, Franklin Lakes, NJ, USA) for 7 days at 21C. Approximately 5×5 mm mycelial plugs excised at the colony periphery, each weighing approximately 100 mg, were used for DNA extraction. One mycelial plug and 500 µl of buffer AP1 (Qiagen, Valencia, CA, USA) were added to a Lysing matrix D tube (MP Biomedicals, Solon OH, USA) and subjected to homogenization at a speed setting of 6 meter/sec for 40 sec using the FastPrep-24 automated cell disruptor (MP Biomedicals). The homogenate was incubated for 10 min at 65°C and processed following the DNeasy plant tissue mini protocol (Qiagen). We selected seven polymorphic SSR markers [32] that were previously identified as polymorphic with the NA1 lineage: PrMS39a, PrMS39b, PrMS43a, PrMS43b, PrMS45 [51], locus 18 and locus 64 [28]. To evaluate the extent of differentiation among populations, PHIst, an appropriate substitute for the fixation index *F*st in asexually reproducing species [52] was estimated using Arlequin ver. 3.1 [53].

### Pathogenicity test

In addition to the 45 isolates used, we also included Pr-102 from oak whose genome has been completely sequenced [54], and Pr-52, a *Rhododendron catawbiense* isolate widely used in inoculation experiments [24], [55], [56], [57]. This experiment started in June 2004, and the majority of isolates employed had been isolated within 12 months. Eighteen-month-old coast live oak seedlings (North Coast Native Nursery, Petaluma, CA) in circular pots (6.5×25 cm) were inoculated with 47 *P. ramorum* isolates (Table 1, set 1) in the glasshouse. There were 10 replicate seedlings per isolate and control. The average minimum and maximum temperatures for the duration of the experiment were 16 and 22°C. All lateral shoots were pruned 6 weeks prior to inoculation.

A sterile scalpel was used to cut a bark-flap, about 6 mm long and 4 mm wide, upwards at 15 cm above the potting medium surface on each stem. A 5 mm diameter agar disc, cut from the margin of a 14-day-old culture growing on 0.5× V8A (100 ml nonclarified V8 juice and 17.5 g of agar/liter), was inserted mycelium-side-down under the flap, the flap closed and the wound sealed with Parafilm and silver Nashua tape (Tyco Adhesives, Franklin, MA). Controls were inoculated with sterile 0.5× V8A discs. The average stem diameter at the site of inoculation was 3.8 mm (range 1.4–6.7 mm). Sixteen days after inoculation, stems were cut at the root collar and the outer bark was carefully scraped back with a sterile scalpel blade at the site of inoculation to expose the maximum extent of any lesion that may have developed. The acropetal and basipetal lengths of the lesion present in the phloem were measured. A small piece of phloem tissue cut from the margins of the longitudinal lesion and the site of inoculation was plated onto P_10_ARP [58] to confirm the presence of *P. ramorum*. Association between lesion size and sample groups were evaluated by Kruskal-Wallis rank sum test implemented in statistical software R 2.7.1 (http://bioconductor.org).

### Evaluation of colony phenotypes

The early senescence phenotype was evaluated for isolates in set 1 (Table 1) in June 2005. Each isolate was inoculated to a solid 0.5× CV8A, with four replicates, and incubated at 20°C for 4 days after which plates were transferred to 28°C for 3 weeks. Isolates were scored as displaying “early senescence phenotype” when at least one of the four duplicates showed growth arrest. Senescence was confirmed by replating isolates that did not grow onto P_10_ARP and incubating at 20°C for a further 3 weeks. For colony morphological phenotypes, *P. ramorum* isolates in sets 1 and 2 (Table 1) were grown on 1× CV8A at 21°C for 7 days and colony patterns were observed. Colonies that had a uniform growth pattern were scored as ‘wild type’ (*wt*) and those with an irregular growth pattern or growth rates slower than *wt* by at least 25% of the average linear growth rate of *wt* were scored as ‘non-wild type’ (*nwt*) [33], [34].

### Microarray design

The *P. ramorum* whole-genome expression oligonucleotide 60-mer arrays (4×72K multiplex format, Roche NimbleGen, Madison, WI, USA) were designed by the manufacturer on the basis of 16,162 gene models derived from the *P. ramorum* database at DOE Joint Genome Institute (transcript.FM_Phyra1_1.fasta.gz; http://genome.jgi-psf.org/Phyra1_1/Phyra1_1.download.ftp.html) and Virginia Bioinformatics Institute (http://vmd.vbi.vt.edu/download/data/prTranscript2.0Annot). On average four probes per open reading frame (ORF), a total of 61,963 probes for 15,488 gene models, were synthesized on each of the four arrays on the multiplex array slide.

### Growth conditions and RNA extraction for microarray analysis

Archival isolates were inoculated on 1× CV8A medium and grown for 7 to 14 days at 21°C in dark. A small mycelial plug obtained at growing colony periphery was then transferred to each of the 60 mm×15 mm Falcon Petri dishes (catalog no. 351007, BD Bioscience, Franklin Lakes, NJ, USA) containing 7 ml of 1× CV8A overlaid with a polycarbonate membrane filter (catalog no. 28157-927; VWR, Brisbane, CA, USA), and grown for 7 days at 21°C under constant cool white fluorescent light at 3.4 µmol m^−2^ s^−1^, as a precaution to avoid experimental noise due to circadian rhythm. Samples were cut into quarters, lifted from the polycarbonate membrane surface; each was transferred to a 2 ml screw-cap microcentrifuge tube (catalog no. 72.694.996 Sarstedt; Fisher Scientific, Santa Clara, CA, USA) and immediately snap-frozen in liquid nitrogen. Each quarter weighed approximately 100 mg and was kept at −80°C until needed. Lysing Matrix A (MP Biomedicals) chilled at −20°C was added to each of the frozen samples. Cells were then disrupted twice for 40 sec using a FastPrep-24 automated cell disruptor, set at 6 meters/sec for 40 sec, in a CoolPrep adapter filled with crushed dry ice. One ml of TRIzol Reagent (Invitrogen Life Technologies) was added to the pulverized sample, and total RNA was extracted according to the TRIzol manufacturer's protocol. Up to 100 µg of total RNA was further cleaned using the RNeasy mini protocol for RNA cleanup (Qiagen, Valencia, CA, USA). RNA purity and concentration were determined by measurements on a NanoDrop 1000 spectrophotometer (Thermo Scientific, Waltham, MA, USA). Finally, integrity of RNA was assessed using RNA Quality Indicator (RQI) values presented by Experion automated electrophoresis system (Bio-Rad, Hercules, CA, USA) [59]. Only high-integrity RNA samples (RQI>9.4) were used for microarray experiments.

### cDNA synthesis, hybridization and image acquisition

cDNA synthesis, labeling, hybridization procedure, data acquisition and normalization were carried out according to the manufacturer's instructions (Roche NimbleGen). Briefly, 10 µg of total RNA and oligo dT primer were used to synthesize the first strand of cDNA, which was followed by the synthesis of the second strand of cDNA to yield double stranded cDNAs. The Cy3 cyanine dye-labeled random 9-mers were then used to label cDNAs a step followed by isopropanol precipitation, vacuumed drying, and hybridization. Hybridization was done at 42°C for 16 to 20 hours on a MAUI Hybridization System (BioMicro Systems, Salt Lake City, UT, USA). After three steps of washing, microarrays were scanned on an Axon GenePix 4000B (Molecular Devices, Sunnyvale, CA, USA). Quantile normalization and background correction across arrays were performed using Robust Multi-chip Average (RMA) algorithm [60] implemented in NimbleScan Version 2.5 software. A MIAME-compliant microarray dataset [61] has been deposited in Filamentous Fungal Gene Expression Database at Yale University (http://bioinfo.townsend.yale.edu/) [62] and in NCBI GEO database (accession number GSE36178). Dataset S1 lists normalized mRNA profiling results and functional annotations.

### Microarray data analysis

Six and seven isolates derived from bay laurel and oak, respectively, were subjected to microarray analysis. No biological or technical replicates for each isolate were performed so as to maximize the number of isolates for comparison and to account for inherent biological variability among isolates [63]. Normalized intensity data for 15,488 genes across 13 arrays were obtained by RMA. In order to minimize noises arising from non-specific hybridization, genes with weak hybridization signals were then removed. First, an intensity ratio of each gene was plotted against the average intensity of gene expression between pairwise samples to visualize intensity-dependent ratio (MA plot, [64]). Second, genes with average hybridization intensity below 64 were removed from the dataset because background noise for these genes was disproportionally high. The remaining 12,516 genes were used for further analysis. Pearson's correlation coefficient between global mRNA expression patterns was then used to cluster cDNA samples using the hclust function with the single linkage option in the statistical software R 2.7.1. Due to the large heterogeneity in expression patterns observed in oak isolates, a fivefold change criterion was used to detect genes that were differentially expressed between groups without using a p-value to seek consistency.

### Analysis of derepressed transposable elements

Derepressed TEs in oak isolates were grouped according to 1) DNA sequence similarity and 2) mRNA expression patterns. 1) BLASTCLUST (BLAST Basic Local Alignment Search Tool, http://www.ncbi.nlm.nih.gov/Ftp/) [65] was used to cluster DNA sequences of TEs with the following parameters: -S (Blast score identity) 0.9, -L (minimum length coverage) 0.5. 2) mRNA profiles of TEs were clustered and visualized using Hierarchical Clustering Explorer [66], with Pearson's correlation coefficient and single linkage options. Blast score identities were then changed until composition of clades changed, in order to empirically determine levels of similarity within and between clades.

### Growth conditions and RNA extraction of axenic cultures for real-time quantitative PCR (qRT-PCR)

qRT-PCR was performed on three selected genes, including one control gene, to validate the microarray experiments on a larger sample size (Table 1). Growth conditions for qRT-PCR were identical to those for microarray mRNA profiling, except that a polycarbonate membrane filter was not used. One 5 mm×5 mm mycelial plug weighing approximately 100 mg was excised from the edges of active growingly colonies and snap-frozen in liquid nitrogen and stored at −80°C until required. The frozen mycelial plug and 500 µl of RNeasy RLT buffer (Qiagen) were added to a Lysing matrix E tube (MP Biomedicals) and the mixture was homogenized at 6 meter/sec for 30 sec twice on a QuickPrep adaptor (MP Biomedicals) using the FastPrep-24 cell disruptor. Total RNA was then extracted according to the manufacturer's protocol for Mini RNeasy Plant and Fungi protocol. Although it is known that adding extraction buffer to frozen samples can alter mRNA profiles, this effect is negligible for *Phytophthora* species [67]. RNA purity and concentration were determined by NanoDrop 1000 spectrophotometer (Thermo Scientific, Waltham, MA, USA).

Genes for which microarray expression was statistically different (t-test with p<0.01) between four TDP and nine non-TDP isolates were screened to identify candidate marker genes. The crinkler gene PR_72359, (hereafter termed *prCRN7*) showed high expression in group A, and a copia-like transposon PR_49580 (hereafter termed *prCopia1*) showed high expression in group B, and were both chosen as markers (Fig. 3 A, G). In order to find a reference gene to normalize the qRT-PCR results, the top 500 candidates genes, showing the smallest coefficient of variation (standard deviation/mean, CV<0.102) in the microarray experiment were screened and Pr_76099 (hereafter termed *EC1*, Fig. 3 C), a gene, which did not display circadian oscillation in expression levels (CV<0.05 in a circadian microarray experiment, data not shown) was selected. Oligonucleotide primers (Table S5) to amplify 100–200 bp of these three genes were designed using Primer3Plus software [68]. qRT-PCR was performed on a Stratagene Mx3000P thermal cycler (Agilent Technologies, Santa Clara, CA, USA) using the Brilliant II SYBR Green QRT-PCR Master Mix Kit, 1-Step (Agilent). Each 20 µl reaction included 10–40 ng/µl of total RNA and 100 nM of each gene-specific primer. All samples, including negative controls, were run in triplicate. Cycling conditions were as follows: 50°C for 30 min, 95°C for 10 min, then 40 cycles of 95°C for 30 sec, and 60°C for 1 min. Expression levels were quantified based on values of the threshold cycles for each sample (Ct). Ct data were analyzed using MxPro version 4.10 software (Agilent). For relative quantification, Ct values for the sequence strain Pr-102 were used as references and the 2^−ΔΔCT^ method was used [69]. 2^−ΔΔCT^ values for *prCopia1* were offset by 16 in order to make the values positive for presentation purposes.

## Supporting Information

Figure S1 **Colony types of NA1 lineage isolates of Californian** ***P. ramorum*** **on clarified V8 medium.** Isolates are a) Pr-102, b) BS-92, c) Pr240, d) Pr-16, e) HC73-5 and f) Pr-177. Note the variation in colony patterns and growth rates as noted by Brasier et al., 2006; *wt*: wild type colonies, *nwt*: non-wild type colonies, senes: early senescence phenotype.
(TIF)

Figure S2 **Hierarchical clustering of TEs expressed in Transposon-derepressed oak isolates.** A total of 297 TEs were clustered based on their expression profiles across the 13 isolates. Each gene's expression values were standardized to have mean zero and standard deviation of one across the 13 isolates. The lighter grey tone in the cluster dendrogram is correlated with a higher expression level. Three distinct clusters, HC1, HC2 and HC3 were selected. From the left lanes are MK79j, MK106, MK548, MK558, MK649a, MK516d, MK649b, HC67-22, HC73-5, Pr-35, MK516a, Pr-102 and Pr-16.
(TIF)

Figure S3 **Two dimensional representation of qRT-PCR markers,** ***prCopia1*** **and** ***prCRN7*** **.** The y and x-axes displays the log2 fold-difference in mean expression values between the reference strain Pr-102 (top left corner) and Californian isolates of *P. ramorum* (−ΔΔCT). For both markers, negative expression values were set to 0. Originating host species are: green, coast live oak; red, tanoak; black, California bay laurel; and turquoise, other hosts. Circle and triangle indicate *wt* and *nwt* colony types, respectively. For *prCopia1*, because the standard Pr-102 had the highest expression level, its expression was offset by 16 for presentation purpose.
(TIF)

Table S1 Mortality of *Phytophthora ramorum* cultures isolated between 2000 and 2002 are summarized. A high rate of death among isolates originating from coast live oak is evident.
(PDF)

Table S2 Californian isolates of *Phytophthora ramorum* used in this study. Source, geographical location, year of isolation, as well as qRT-PCR data for *prCopia1* and *prCRN7* genes, colony phenotypes and SSR multilocus genotype for each isolate are shown.
(PDF)

Table S3 AMOVA results for geographic populations of *Phytophthora ramorum*. A significant genetic differentiation (p<0.001) was detected in SSR multilocus genotype data among the three populations, namely, Monterey, Santa Cruz and Sonoma.
(PDF)

Table S4 AMOVA results for *Phytophthora ramorum* populations based on originating host species. Population structure was not detected (p = 0.62) in multilocus SSR genotypes among isolates originating from tanoak, coast live oak and bay laurel.
(PDF)

Table S5 Oligonucleotide primers used for qRT-PCR analysis of Californian isolates of *Phytophthora ramorum*.
(PDF)

Dataset S1 This dataset provides mRNA profiling results and information for each gene such as functional annotation, GO terms, transposon class and expression clusters for 12,516 detected genes by means of *Phytophthora ramorum* NimbleGen expression microarray. Each of columns is explained in the attached worksheet “Readme”.
(XLS)
