# Supplementary figures and images for: Phenotypic Diversification Is Associated with Host-Induced Transposon Derepression in the Sudden Oak Death Pathogen Phytophthora ramorum
Source: PLoS One. 2012 Apr 18;7(4):e34728. doi: 10.1371/journal.pone.0034728 (PMC3329494; doi:10.1371/journal.pone.0034728)

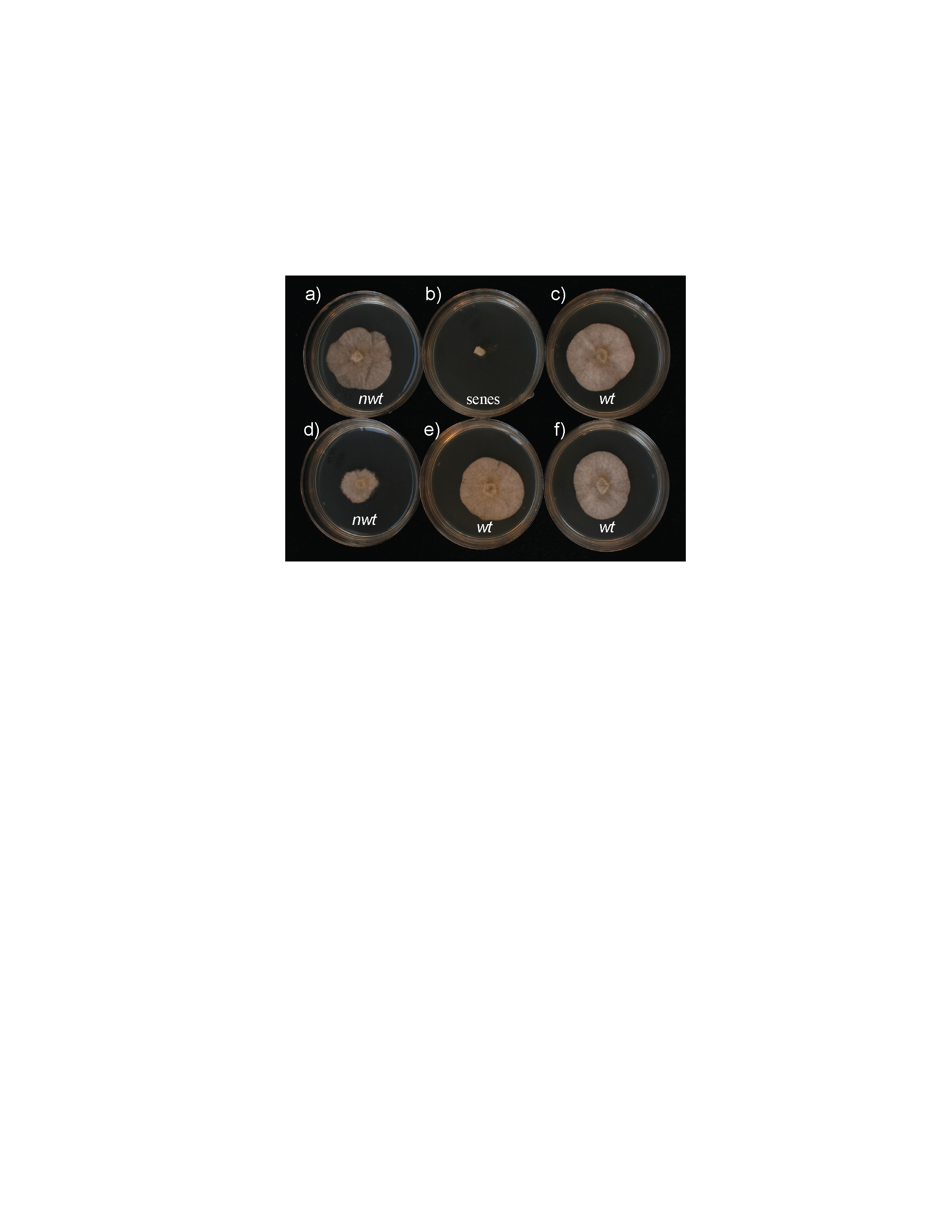

Supplement: Figure S1 — Colony types of NA1 lineage isolates of Californian P. ramorum on clarified V8 medium. Isolates are a) Pr-102, b) BS-92, c) Pr240, d) Pr-16, e) HC73-5 and f) Pr-177. Note the variation in colony patterns and growth rates as noted by Brasier et al., 2006; wt: wild type colonies, nwt: non-wild type colonies, senes: early senescence phenotype. (TIF) [file pone.0034728.s001.tif]

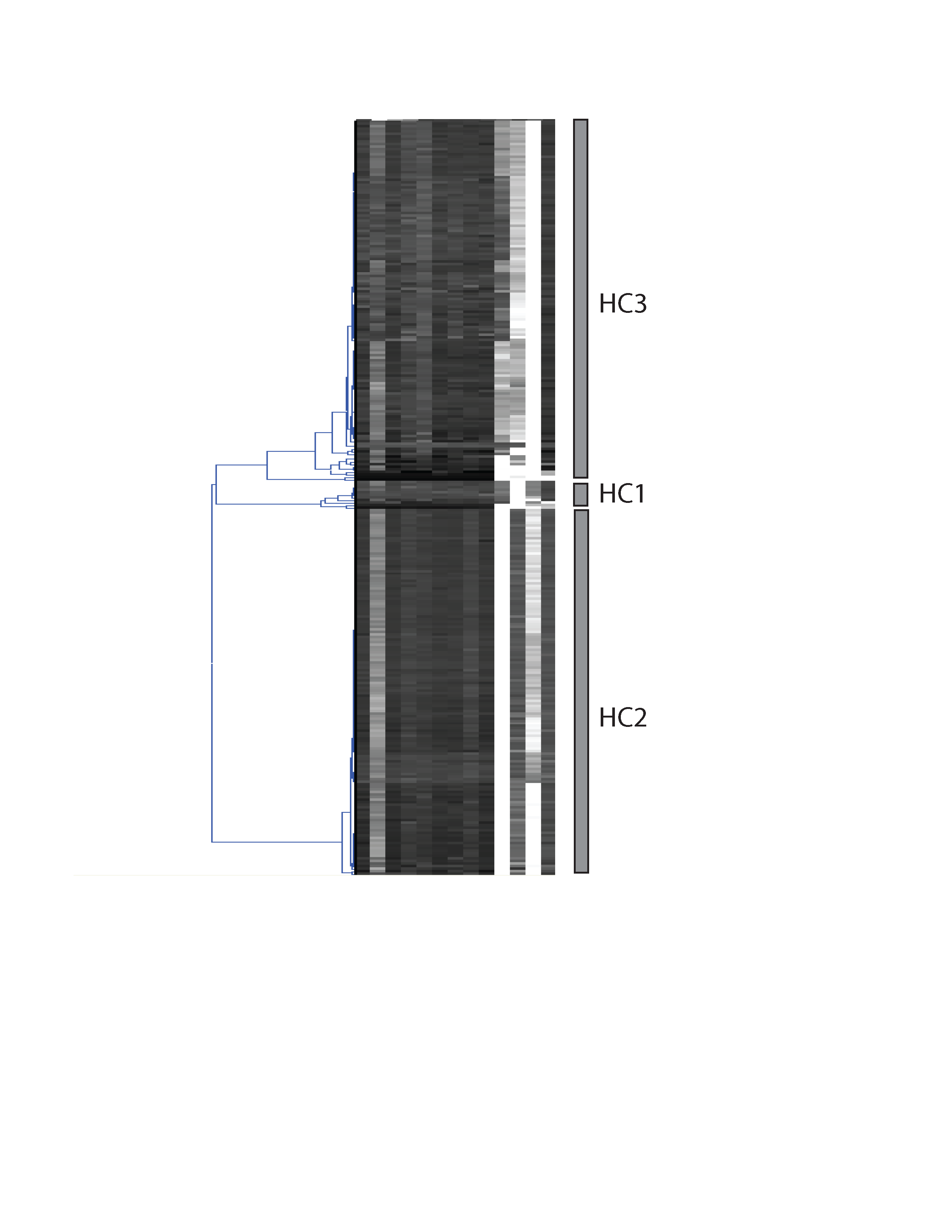

Supplement: Figure S2 — Hierarchical clustering of TEs expressed in Transposon-derepressed oak isolates. A total of 297 TEs were clustered based on their expression profiles across the 13 isolates. Each gene's expression values were standardized to have mean zero and standard deviation of one across the 13 isolates. The lighter grey tone in the cluster dendrogram is correlated with a higher expression level. Three distinct clusters, HC1, HC2 and HC3 were selected. From the left lanes are MK79j, MK106, MK548, MK558, MK649a, MK516d, MK649b, HC67-22, HC73-5, Pr-35, MK516a, Pr-102 and Pr-16. (TIF) [file pone.0034728.s002.tif]

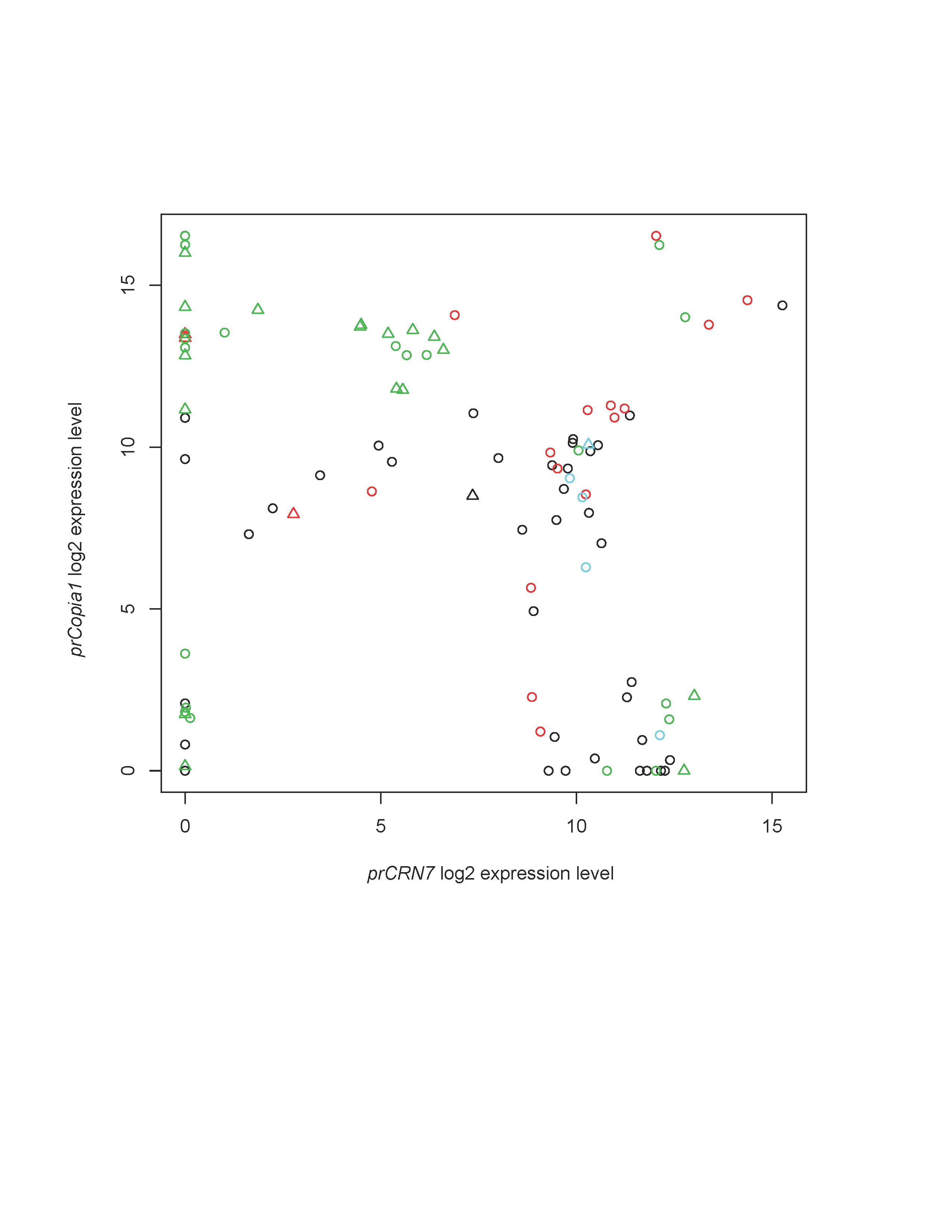

Supplement: Figure S3 — Two dimensional representation of qRT-PCR markers, prCopia1 and prCRN7 . The y and x-axes displays the log2 fold-difference in mean expression values between the reference strain Pr-102 (top left corner) and Californian isolates of P. ramorum (−ΔΔCT). For both markers, negative expression values were set to 0. Originating host species are: green, coast live oak; red, tanoak; black, California bay laurel; and turquoise, other hosts. Circle and triangle indicate wt and nwt colony types, respectively. For prCopia1, because the standard Pr-102 had the highest expression level, its expression was offset by 16 for presentation purpose. (TIF) [file pone.0034728.s003.tif]
