# Supplementary material for: Phenotypic Diversification Is Associated with Host-Induced Transposon Derepression in the Sudden Oak Death Pathogen Phytophthora ramorum
Source: PLoS One. 2012 Apr 18;7(4):e34728. doi: 10.1371/journal.pone.0034728 (PMC3329494; doi:10.1371/journal.pone.0034728)
Supplement: Table S1 — Mortality of Phytophthora ramorum cultures isolated between 2000 and 2002 are summarized. A high rate of death among isolates originating from coast live oak is evident. (PDF) [file pone.0034728.s004.pdf]

Table S1. The death toll of *P. ramorum* isolated between 2000 and 2002. A high rate of death among isolates originating from coast live is evident.

Host species	common name of host	total number of isolates	Dead islates[1]		
			Observed	Expected	P-value
Notholitocarpus densiflorus	tanoak	50	24	25.5	1.01E+00
Quercus agrifolia	coast live oak	60	43	30.6	1.48E-03
Quercus kelloggii	California black oak	2	2	1.0	9.96E-01
Quercus parvula v. shrevei	Shreve oak	1	0	0.5	1.31E+00
Rhododendron spp.	Rhododendron	9	4	4.6	8.50E-01
Umbellularia californica	California bay laurel	61	20	31.1	2.90E-03
Vaccinium ovatum	California huckleberry	9	4	4.6	7.44E-01
Others		8	5	4.1	9.61E-01

1 Observed number of dead isolates and expected number of dead isolates if probabilities of death are independent of host species.

2 P-values were determined using Fisher's exact test with Benjamini and Hochberg multiple testing correction.
