# Supplementary material for: Phenotypic Diversification Is Associated with Host-Induced Transposon Derepression in the Sudden Oak Death Pathogen Phytophthora ramorum
Source: PLoS One. 2012 Apr 18;7(4):e34728. doi: 10.1371/journal.pone.0034728 (PMC3329494; doi:10.1371/journal.pone.0034728)
Supplement: Table S2 — Californian isolates of Phytophthora ramorum used in this study. Source, geographical location, year of isolation, as well as qRT-PCR data for prCopia1 and prCRN7 genes, colony phenotypes and SSR multilocus genotype for each isolate are shown. (PDF) [file pone.0034728.s005.pdf]

Table S2. Californian isolates of *Phytophthora ramorum* used in this study.

Isolate numbers	Source	County	Year	ddCt cpCRN7	ddCt prCopia1	Colony type	Senescence	MLG*
**set 1**								
BS-29	*Notholithocarpus densiflorus*	Monterey	2004	9.3	9.8	wt	No	504
BS-34	*Notholithocarpus densiflorus*	Monterey	2004	0.0	13.5	nwt	No	515
BS-57	*Quercus agrifolia*	Monterey	2004	4.5	13.7	nwt	No	CBD
BS-67	*Umbellularia californica*	Monterey	2004	15.3	14.4	wt	Yes	515
BS-73	*Notholithocarpus densiflorus*	Monterey	2004	13.4	13.8	wt	Yes	514
BS-77	*Notholithocarpus densiflorus*	Monterey	2004	14.4	14.5	wt	No	515
BS-78	*Umbellularia californica*	Monterey	2004	8.0	9.7	wt	No	515
BS-81	*Notholithocarpus densiflorus*	Monterey	2004	4.8	8.6	wt	Yes	515
BS-87	*Umbellularia californica*	Monterey	2004	11.4	11.0	wt	No	523
BS-90	*Umbellularia californica*	Monterey	2004	7.3	8.5	nwt	Yes	515
BS-92	*Quercus agrifolia*	Monterey	2004	0.0	12.8	nwt	Yes	515
BS-94	*Quercus agrifolia*	Monterey	2004	5.7	12.8	wt	Yes	509
BS-95	*Quercus agrifolia*	Monterey	2004	4.5	13.8	nwt	No	515
BS-96	*Umbellularia californica*	Monterey	2004	9.7	8.7	wt	No	513
HC65-12	*Umbellularia californica*	Santa Cruz	2004	9.9	10.2	wt	No	516
HC65-13	*Notholithocarpus densiflorus*	Santa Cruz	2004	10.9	11.3	wt	No	520
HC67-22	*Umbellularia californica*	Santa Cruz	2004	10.4	9.9	wt	No	520
HC69-28	*Notholithocarpus densiflorus*	Santa Cruz	2004	11.2	11.2	wt	No	522
HC72-12	*Notholithocarpus densiflorus*	Santa Cruz	2004	11.0	10.9	wt	No	503
HC73-17	*Notholithocarpus densiflorus*	Santa Cruz	2004	9.5	9.3	wt	No	519
HC73-4	*Umbellularia californica*	Santa Cruz	2004	9.3	0.0	wt	No	519
HC73-5	*Umbellularia californica*	Santa Cruz	2004	9.9	10.1	wt	No	521
HC73-9	*Quercus agrifolia*	Santa Cruz	2004	10.1	9.9	wt	No	511
HC74-28	*Umbellularia californica*	Santa Cruz	2004	7.4	11.0	wt	No	519
HC80-14	*Notholithocarpus densiflorus*	Santa Cruz	2004	10.3	11.1	wt	No	521
MR-12	*Quercus agrifolia*	Santa Cruz	2004	5.8	13.6	nwt	Yes	519
MR-13	*Quercus agrifolia*	Santa Cruz	2004	6.2	12.8	wt	No	519
MR-14	*Quercus agrifolia*	Santa Cruz	2004	0.0	13.1	wt	Yes	CBD
Pr-12	*Quercus agrifolia*	Monterey	2000	12.8	14.0	wt	Yes	505
Pr-16	*Quercus agrifolia*	Santa Cruz	2000	0.0	14.3	nwt	No	529
Pr-21	*Quercus agrifolia*	Sonoma	2000	6.4	13.4	nwt	Yes	518
Pr-24	*Quercus agrifolia*	Sonoma	2000	6.6	13.0	nwt	No	527
Pr-35	*Quercus agrifolia*	Sonoma	2000	0.0	13.5	wt	Yes	CBD
Pr-37	*Quercus agrifolia*	Sonoma	2000	5.6	11.8	nwt	Yes	510
Pr-108	*Umbellularia californica*	Sonoma	2001	5.3	9.5	wt	No	519
Pr-142	*Umbellularia californica*	Sonoma	2001	0.0	10.9	wt	No	CBD
Pr-143	*Quercus agrifolia*	Sonoma	2001	5.4	11.8	nwt	Yes	518
Pr-177	*Umbellularia californica*	Sonoma	2002	9.5	7.7	wt	No	532
Pr-178	*Umbellularia californica*	Sonoma	2002	8.6	7.4	wt	No	516
Pr-179	*Notholithocarpus densiflorus*	Sonoma	2002	12.0	16.5	wt	No	519
Pr-180	*Notholithocarpus densiflorus*	Sonoma	2002	8.8	5.7	wt	No	523
Pr-181	*Notholithocarpus densiflorus*	Sonoma	2002	0.0	13.4	nwt	Yes	524
Pr-184	*Notholithocarpus densiflorus*	Sonoma	2002	9.1	1.2	wt	No	523
Pr-189	*Notholithocarpus densiflorus*	Sonoma	2002	8.9	2.3	wt	No	519
Pr-240	*Umbellularia californica*	Sonoma	2002	0.0	0.0	wt	No	506
Pr-52	*Rhododendron catawbiense*	Santa Cruz	2000	9.8	9.0	wt	No	527
Pr-102	*Quercus agrifolia*	Marin	2001	0.0	16.0	nwt	Yes	500
**set 2**								
MK106	*Quercus agrifolia*	San Mateo	2008	0.0	1.9	wt	n/a	510
MK548	*Umbellularia californica*	San Mateo	2008	10.5	0.4	wt	n/a	512
MK558	*Quercus agrifolia*	San Mateo	2008	10.8	0.0	wt	n/a	516
MK1365	*Quercus agrifolia*	San Mateo	2009	5.2	13.5	nwt	n/a	516
MK1702	*Umbellularia californica*	San Mateo	2009	0.0	2.1	wt	n/a	519
MK1702A	*Umbellularia californica*	San Mateo	2009	0.0	9.6	wt	n/a	519
MK1856	*Umbellularia californica*	San Mateo	2009	4.9	10.0	wt	n/a	519
MK1869A	*Quercus agrifolia*	San Mateo	2009	0.0	11.2	nwt	n/a	519
MK2331	*Umbellularia californica*	San Mateo	2009	8.9	4.9	wt	n/a	516
MK2338A	*Umbellularia californica*	San Mateo	2009	9.4	1.0	wt	n/a	516
MK116A	*Quercus agrifolia*	San Mateo	2009	5.4	13.1	wt	n/a	511
MK516a	*Quercus agrifolia*	San Mateo	2008	1.9	14.2	nwt	n/a	512
MK516d	*Quercus agrifolia*	San Mateo	2008	0.1	1.6	wt	n/a	512
MK649a	*Umbellularia californica*	San Mateo	2008	10.6	7.0	wt	n/a	516

MK649b	*Umbellularia californica*	San Mateo	2008	12.3	0.0	wt	n/a	512
MK79j	*Umbellularia californica*	San Mateo	2008	12.4	0.3	wt	n/a	510
MKP10A	*Umbellularia californica*	San Mateo	2009	9.7	0.0	wt	n/a	511
Mr1126	*Umbellularia californica*	Marin	2007	11.6	0.0	wt	n/a	N/A
Mr1135	*Umbellularia californica*	Marin	2007	12.2	0.0	wt	n/a	N/A
Mr1140	*Umbellularia californica*	Marin	2007	11.4	2.7	wt	n/a	N/A
Mr1284	*Umbellularia californica*	Marin	2007	11.3	2.3	wt	n/a	N/A
Mr1295	*Umbellularia californica*	Marin	2007	11.8	0.0	wt	n/a	N/A
Mr1322	*Umbellularia californica*	Sonoma	2007	0.0	0.8	wt	n/a	N/A
Mr1323	*Umbellularia californica*	Sonoma	2007	11.7	0.9	wt	n/a	N/A
Mr1336	*Umbellularia californica*	Sonoma	2007	0.0	0.0	wt	n/a	N/A
Pr-1	*Quercus agrifolia*	Marin	2000	13.0	2.3	nwt	n/a	CBD
Pr-2	*Notholithocarpus densiflorus*	Marin	2000	2.8	7.9	nwt	n/a	515
Pr-6	*Quercus agrifolia*	Marin	2000	0.0	1.7	nwt	n/a	517
Pr-8	*Quercus agrifolia*	Napa	2000	12.4	1.6	wt	n/a	530
Pr-10	*Notholithocarpus densiflorus*	Monterey	2000	10.2	8.5	wt	n/a	511
Pr-15	*Notholithocarpus densiflorus*	Santa Cruz	2000	6.9	14.1	wt	n/a	516
Pr-25	*Quercus agrifolia*	Sonoma	2000	0.0	13.3	wt	n/a	515
Pr-26	*Quercus agrifolia*	Marin	2000	1.0	13.5	wt	n/a	CBD
Pr-41	*Quercus agrifolia*	Marin	2000	0.0	3.6	wt	n/a	516
Pr-48	*Quercus agrifolia*	Sonoma	2000	12.0	0.0	wt	n/a	528
Pr-50	*Quercus agrifolia*	Sonoma	2000	12.3	2.1	wt	n/a	509
Pr-53	*Umbellularia californica*	Sonoma	2001	3.4	9.1	wt	n/a	508
Pr-62	*Quercus agrifolia*	San Mateo	2001	0.0	1.8	wt	n/a	502
Pr-68	*Vaccinium ovatum*	Marin	2001	10.2	6.3	wt	n/a	519
Pr-69	*Vaccinium ovatum*	Marin	2001	10.1	8.5	wt	n/a	516
Pr-77	*Umbellularia californica*	Marin	2001	10.5	10.1	wt	n/a	523
Pr-78	*Umbellularia californica*	Marin	2001	10.3	8.0	wt	n/a	519
Pr-88	*Umbellularia californica*	Sonoma	2001	9.4	9.4	wt	n/a	511
Pr-91	*Umbellularia californica*	Santa Cruz	2001	1.6	7.3	wt	n/a	525
Pr-96	*Umbellularia californica*	Marin	2001	9.8	9.3	wt	n/a	531
Pr-98	*Umbellularia californica*	Napa	2001	2.2	8.1	wt	n/a	515
Pr-420	*Quercus agrifolia*	Marin	2005	0.0	0.1	nwt	n/a	525
Pr-461	*Quercus chrysolepsis*	Humboldt	2005	12.1	1.1	wt	n/a	511
Pr-514	stream water	Humboldt	2007	10.3	10.1	nwt	n/a	EU1
Pr-518	*Quercus agrifolia*	Marin	2007	12.8	0.0	nwt	n/a	526
Pr-774	*Quercus agrifolia*	Sonoma	2009	12.1	16.2	wt	n/a	510
Pr-775	*Quercus agrifolia*	Sonoma	2009	0.0	16.5	wt	n/a	510
Pr-776	*Quercus agrifolia*	Sonoma	2009	0.0	16.3	wt	n/a	507
Pr-777	*Quercus agrifolia*	Sonoma	2009	0.0	16.5	wt	n/a	501

* MLG: multilocus genotypes due to SSR markers.
