# Supplementary material for: Phenotypic Diversification Is Associated with Host-Induced Transposon Derepression in the Sudden Oak Death Pathogen Phytophthora ramorum
Source: PLoS One. 2012 Apr 18;7(4):e34728. doi: 10.1371/journal.pone.0034728 (PMC3329494; doi:10.1371/journal.pone.0034728)
Supplement: Table S3 — AMOVA results for geographic populations of Phytophthora ramorum. A significant genetic differentiation (p<0.001) was detected in SSR multilocus genotype data among the three populations, namely, Monterey, Santa Cruz and Sonoma. (PDF) [file pone.0034728.s006.pdf]

Table S3. AMOVA results for geographic populations of *P. ramorum*. A significant genetic differentiation was detected among the three geographical populations, namely Monterey, Santa Cruz and Sonoma.

Source of variation	df	Sum of squares	Variance components	% of variation	Φ_{ST}	P-value
Among all populations	2	13.726	0.187	11.4	0.11425	0.0000
Within populations	89	123.306	1.385	88.6		

AMOVA was conducted on microsatellite multilocus genotypes to measure the genetic differentiation among populations using the program ARLEQUIN (Excoffier and Schneider, 2005). The AMOVA calculates ΦST, an index of population subdivision. The significance of FST was tested by 10,000 permutations. All isolates in Table 1, set1 except for Pr-52, a total of 46 isolates were used. Pr-102 from Malin County was included in the Sonoma County population.
