# Supplementary material for: Phenotypic Diversification Is Associated with Host-Induced Transposon Derepression in the Sudden Oak Death Pathogen Phytophthora ramorum
Source: PLoS One. 2012 Apr 18;7(4):e34728. doi: 10.1371/journal.pone.0034728 (PMC3329494; doi:10.1371/journal.pone.0034728)
Supplement: Table S4 — AMOVA results for Phytophthora ramorum populations based on originating host species. Population structure was not detected (p = 0.62) in multilocus SSR genotypes among isolates originating from tanoak, coast live oak and bay laurel. (PDF) [file pone.0034728.s007.pdf]

Table S4. AMOVA results for *P. ramorum* populations based on originating host species. Population structure was not detected among isolates originating from tanoak, coast live oak and California bay laurel.

Source of variation	df	Sum of squares	Variance components	% of variation	Φ_{ST}	P-value
Among all populations	2	2.614	-0.007	-0.4	-0.00284	0.62762
Within populations	89	134.419	1.510	100.4		

The same set of isolates in Table S3 were used.
