# Supplementary material for: Phenotypic Diversification Is Associated with Host-Induced Transposon Derepression in the Sudden Oak Death Pathogen Phytophthora ramorum
Source: PLoS One. 2012 Apr 18;7(4):e34728. doi: 10.1371/journal.pone.0034728 (PMC3329494; doi:10.1371/journal.pone.0034728)
Supplement: Table S5 — Oligonucleotide primers used for qRT-PCR analysis of Californian isolates of Phytophthora ramorum. (PDF) [file pone.0034728.s008.pdf]

Table S5. Oligonucleotide primers used for qRT-PCR analysis

Locus	Description	Target Gene ID	Forward/Reverse	Product size/bp
**Selected marker genes**				
PrCRN7	Crinkler effector homolog	Pr_72359	TCTACTGGCAAGTGGTGCTG/ CCCAGTCGGGACCTAACATA	173
PrCopia1	Ty1 copia polyprotein	Pr_49580 Pr_39792[1]	CAAAGGGGTACGAGGAAACA/ AGTAGGCGAAACCAAACACG	100
**Selected endogenous control gene**				
EC1	hypothetical protein	Pr_76099	ACCATCACCAAGGGTCTCAG/ TGTCGTACTGCGCAAAATTC	142

[1] DNA similarity between genes for polyproteins Pr_49580 and Pr39792 is 98% and the annealing sites for primers are perfectly conserved.
